# Coetaneous catalytic kinetic resolution of alkynes and azides through asymmetric triazole formation

**DOI:** 10.1038/s41598-019-50940-4

**Published:** 2019-10-21

**Authors:** William D. G. Brittain, Andrew G. Dalling, Zhenquan Sun, Cécile S. Le Duff, Louise Male, Benjamin R. Buckley, John S. Fossey

**Affiliations:** 1 School of Chemistry, University of Birmingham, Edgbaston, Birmingham, West Midlands B15 2TT UK; 2 School of Chemistry and Chemical Engineering, Sun Yat-Sen University, 135 Xingang Rd. W., Guangzhou, 510275 China; 3 NMR Facility, School of Chemistry, University of Birmingham, Edgbaston, Birmingham, B15 2TT West Midlands UK; 4 X-Ray Crystallography Facility, School of Chemistry, University of Birmingham, Edgbaston, Birmingham, West Midlands B15 2TT UK; 5 Department of Chemistry, Loughborough University, Loughborough, Leicestershire LE11 3TU UK

**Keywords:** Asymmetric catalysis, Homogeneous catalysis, Chemistry, Synthetic chemistry methodology, Stereochemistry

## Abstract

A non-enzymatic simultaneous (coined coetaneous) kinetic resolution of a racemic alkyne and racemic azide, utilising an asymmetric CuAAC reaction is reported. The use of a CuCl (*R,R*)-Ph-Pybox catalyst system effects a simultaneous kinetic resolution of two racemic starting materials to give one major triazolic diastereoisomer in the ratio 74:12:4:10 (dr 84:16, 90% *ee* maj). The corresponding control reaction using an achiral copper catalyst gives the four possible diastereoisomers in a 23:27:23:27 ratio, demonstrating minimal inherent substrate control.

## Introduction

The copper-catalysed azide-alkyne cycloaddition (CuAAC) pioneered by both Meldal and Sharpless has become a ubiquitous molecular fragment-linking reaction^1–3^. The product 1,4-substituted, 1,2,3-triazoles, along with their alkyne and azide building blocks, in enantioenriched forms are important motifs^4–9^. Triazoles for example, have become crucial to research arenas including fragment based drug discovery (FBDD) and supramolecular chemistry^10^. Whilst scalemic alkynes and azides are important building blocks for a myriad of transformations^4,5^.

**Scheme 1:**
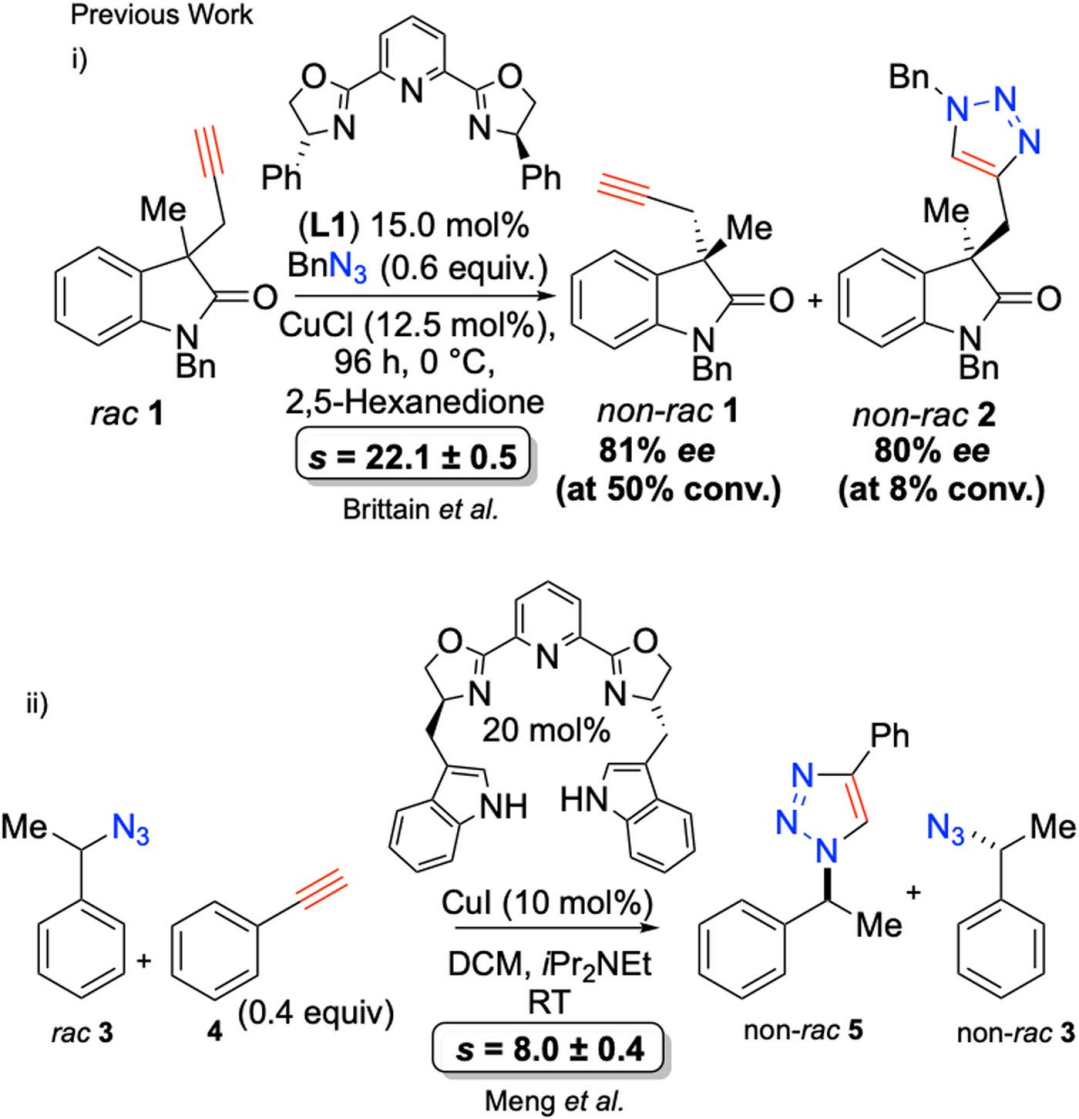
Previous Work: (i) Kinetic Resolution of Alkynes by Brittain *et al*.^18^. (ii) Kinetic Resolution of Azides by Meng *et al*.^20^.

Catalytic kinetic resolution (KR) occurs when one enantiomer of a racemic substrate is preferentially activated towards reaction by a chiral catalyst (through competing diastereomeric transition states), leading to more rapid formation of one enantiomer of product. At 50% conversion of starting racemic material, effective catalytic KR will have occurred if high *ee* of product and high *ee* of unreacted starting material are obtained. The effectiveness of a kinetic resolution may be judged by a criterion named selectivity factor (*s*). Selectivity factor is the ratio of the rate constants for reaction of each enantiomer in a given asymmetric transformation^11^. Enzymes are capable of performing catalytic KR, albeit under a narrow range of conditions with limited substrate scope^12,13^. Kinetic resolution has been widely studied^11^. Fu and co-workers have championed catalytic KR, applying planar chiral DMAP-derivative catalysts to the successful KR of secondary alcohols^14–16^. Catalytic KR has also been successfully employed in copper-catalysed azide-alkyne cycloadditions leading to enantioenriched chiral triazoles and the recovery of enantioenriched starting materials (Scheme 1i and ii)^17–21^, and complete consumption of starting materials in the case of dynamic kinetic resolution^22^. Desymmetrisation by asymmetric triazole formation has also been successfully achieved^23–25^.

**Scheme 2:**
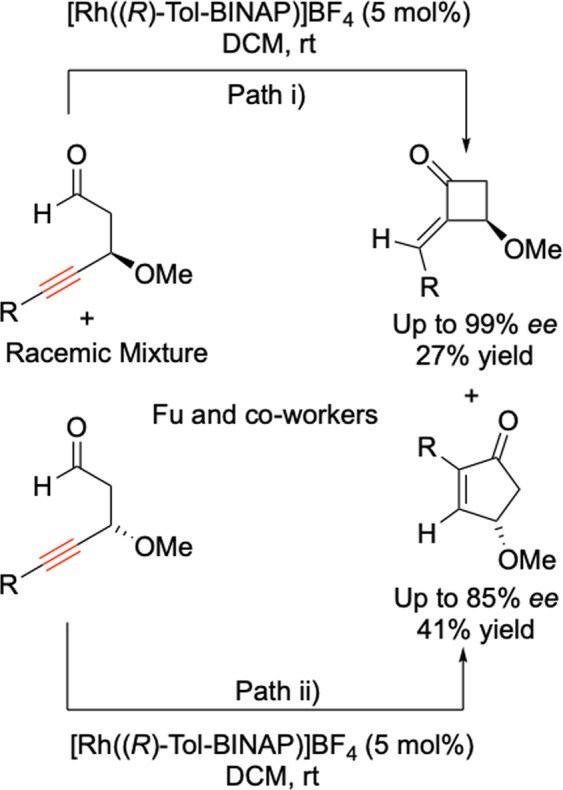
*Parallel Kinetic Resolution of 4-Alkynals, Fu and co-workers*^30^ Path (i) Reaction of enantiomer (*R*) to form cyclobutaneone product. Path (ii) Reaction of enantiomer (*S*) to form cyclopentenone product.

Parallel kinetic resolution is a well-established field, where a single chiral starting materials’ enantiomers undergo simultaneous divergent asymmetric transformations yielding different enantioenriched products from either enantiomer of starting material^26–29^. For example Fu and co-workers utilised parallel kinetic resolution to resolve 4-alkynals (Scheme 2)^30^.

Herein, we investigate a simultaneous, rather than parallel, kinetic resolution of two racemic substrates, under control of a single chiral catalyst, and coin the term coetaneous resolution to describe it. Upon coetaneous resolution of two racemic substrates, the ideal scenario would be formation, at 50% conversion, of a single diastereoisomer of the product formed from one enantiomer of each substrate. This ideal process would leave the opposite enantiomers of the substrates unreacted in high enantiopurity. Thus, simultaneously kinetically resolving two chiral starting materials under one reaction and product manifold.

**Figure 1:**
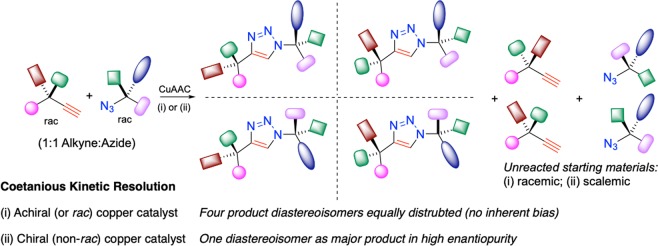
Principle of Coetaneous Kinetic Resolution: (i) the scenario where an achiral catalyst delivers inherent equimolar distribution of diastereoisomers; (ii) the scenario where a chiral catalyst selectively reacts with starting materials.

We chose to focus on the CuAAC of racemic substrates, such that, under catalyst control, the starting materials (chiral azides and alkynes) could react selectively to give a major stereoisomer, among the four possible expected diastereomeric triazole products. Conversely, in the case where no substrate control exists, the use of an achiral catalyst will lead to equal consumption of starting material enantiomers and delivery of an equimolar distribution of the four stereoisomers of product (Fig. 1). Asymmetric synthesis using two catalysts to control the formation of two stereogenic centres has been elegantly demonstrated by Carreira and co-workers. See refs^26,27,^^31,32^.

## Results and Discussion

Based on previous work on catalytic kinetic resolution of alkynes and azides, we chose the PyBox ligand family for creation of a chiral copper catalyst and selected (*R,R*)-Ph-PyBox as a suitable ligand for our investigations^20,25,33,34^. To investigate the potential for coetaneous catalytic kinetic resolution we focused on substrates with demonstrable efficacy in *standard* catalytic kinetic resolutions. Quaternary oxindole **1** (as employed in Scheme 1i), was selected as the alkyne-containing component. Oxindoles are important, biologically-relevant, scaffolds having found wide application, including as calcium channel blockers^35^, anti-angiogenics^36^, antitumour agents^37–39^ and analgesics^35^. Azide **3** (as employed in Scheme 1ii) was chosen owing to its previously reported application in the first catalytic kinetic, copper-catalysed triazole forming, resolution by Fokin and co-workers^20^.

We have previously explored the selectivity of alkyne **1** towards kinetic resolution and found an intriguing solvent dependency upon selectivity^18^, however resolution of azide **3** had not been employed under those same conditions (shown in Scheme 1i). In order to probe this azide **3** was reacted with 0.5 equivalents of phenyl acetylene, 15 mol% **L1** and CuCl (12.5 mol%) in acetone-*d*_6_ and the reaction progress was monitored *in-situ via* proton nuclear magnetic resonance (^1^H NMR) spectroscopy.

From the resolution of azide **3** in acetone a selectivity factor of *s* = 7.4 was determined (Table 1, entry 1). Since, resolution of alkyne **1** had been shown to be superior, in a previous study, when 2,5-hexanedione had been employed as reaction solvent the resolution of **3** was repeated using this dione solvent (unlocked ^1^H NMR spectroscopy reaction monitoring, see ESI) which gave conversions and selectivity in line with that when acetone was used as solvent (*s* = 7.1), (Table 1, entries 1 and 2 *versus* 3 and 4) suggesting that the dione solvent-effect is manifest primarily in alkyne rather than azide selectivity.

**Table 1:** Kinetic resolution of alkyne 1 and azide 3 under the same reaction conditions.


Entry	R^1^	R^2^	Solvent	Selectivity Factor (*s*)
1	Ph (**4**)	C_6_H_5_CHCH_3_(**3**)	Acetone	7.4
2	Ph (**4**)	C_6_H_5_CHCH_3_(**3**)	2,5-Hexanedione	7.1
3	Oxindole (**1**)	Bn	Acetone	5.3^a^
4	Oxindole (**1**)	Bn	2,5-Hexanedione	22.1^a^

Reaction was carried out with 0.6 equiv. non-chiral material to 1 equiv. chiral substrate. ^a^Selectivity is that which has been previously reported by Brittain *et al*.^17^.

**Scheme 3:**
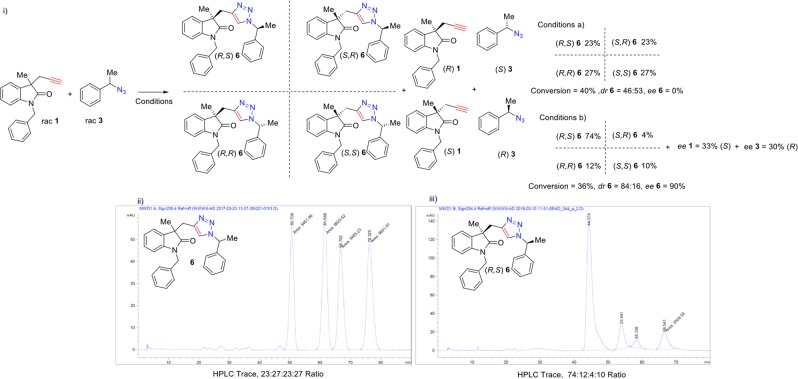
(i) Coetaneous Kinetic Resolution of Azides and Alkynes. Conversion was determined by ^1^H NMR spectroscopy. Enantioenrichment of **6** and **1** was determined by HPLC using a chiral stationary phase. “Stereochemical nomenclature to describe diastereoisomers as follows: The first letter inside the brackets refers to the stereochemical descriptor at the oxindole stereogenic centre, the second letter within the brackets refers to stereochemical descriptor at the stereogenic centre adjacent to the triazole ring”. Enantioenrichment of azide **3** was determined by GC using a chiral stationary phase. (ii) HPLC trace of outcomes using conditions: (**a**) Alkyne **1** (1 equiv.) to azide **3** (1 equiv.), TBTA (15.0 mol%), CuCl (12.5 mol%) in 2,5-hexanedione at 0 °C for 96 h. (iii) HPLC trace using conditions; or (**b**) Alkyne **1** (1 equiv.) to azide **3** (1 equiv.), CuCl (12.5 mol%), **L1** (15.0 mol%) in 2,5-hexanedione at 0 °C for 96 h.

The inherent diastereoselectivity of the CuAAC of a reaction of **1** with **3** was probed (Scheme 3i), to determine any contribution to diastereoselectivity from substrate bias. Compounds **1** and **3** were reacted together with tris[(1-benzyl-1*H*-1,2,3-triazol-4-yl)methyl]amine (TBTA) as an achiral ligand giving a product diastereoisomer ratio of 23:27:23:27 *via* HPLC, thus demonstrating there was little inherent diastereoselectivity between the two substrates in the CuAAC reaction (Scheme 3ii).

The coetaneous kinetic resolution of **1** and **3** was then attempted. To our delight, it was found that a mixture of 1:1 of **3** and **1** in the presence of 15 mol% **L1** and 12.5 mol% CuCl catalyst provided a 74:12:4:10 diastereoisomeric ratio of product **6** (Scheme 3iii). This showed that indeed the reaction successfully resolved the two starting materials simultaneously.

To enable comparison of the selectivity for each enantiomer in this diastereo- and enantio- selective process the selectivity factors for each enantiomer of alkyne **1** and azide **3** were determined as follows. Using enantiopure substrates as the limiting reagent the selectivity towards that component could be analysed (Table 2). The absolute configuration of **1** was evidenced by X-ray diffractometry of a single crystal of an iodo alkyne derived from one enantiomer of enantiopure alkyne **1** (see compound **7** ESI) and the absolute configuration of **3** by comparison to it and by employing in the synthesis literature data and protocols (see ESI). Thus, also allowing for assignment of the absolute stereochemistry of the products (**6**, as noted in Scheme 3, and see ESI).

**Table 2:** Kinetic resolution with enantiopure partners.


Entry	Alkyne Stereochemistry^a^	Azide Stereochemistry^b^	Conv.^c^	*SM ee* (%)^d^	*s*
1	*Rac*	(*S*)	33	35	8.3
2	*Rac*	(*R*)	14	8	3.2
3	(*S*)	*Rac*	29	5	1.4
4	(*R*)	*Rac*	30	14	2.2

Reactions were run with 0.5 equiv. of single enantiomer substrate to 1 equiv. of racemic substrate. ^a^Enantiopure alkyne **1** was obtained by preparative chiral HPLC (see ESI). ^b^Enantiopure azide **3** was synthesised according to literature procedure (see ESI). ^c^Conversion was determined by integration of ^1^H NMR spectra (see ESI). ^d^SM refers to the recovered alkyne/azide following resolution (*rac* component before resolution).

From the data presented in Table 2 it can be seen that consumption of racemic alkyne is faster and more selective (in a reaction catalysed by a catalyst derived from **L1**) in combination with (*S*) azide. As well as the observation that consumption of racemic azide is essentially equally rapid in combination with either enantiomer of alkyne (in a reaction catalysed by a catalyst derived from **L1**), with only a slight difference in selectivity, it being subtly better with (*R*) alkyne. These, and earlier, data reveal two important features, firstly issues of selectivity are more dependent upon alkyne than azide stereochemistry (in this example) and secondly that across these four experiments with a single enantiomer component the faster reacting and more selective examples involve (*S*) azide and (*R*) alkyne, which corresponds to the major product (of Scheme 3 iii) being formed from these same two enantiomers. Thus, adding support for the hypothesis that a coetaneous kinetic resolution is taking place.

## Conclusions

These preliminary findings are to the best of our knowledge the first non-enzymatic example of two racemic starting materials being successfully kinetically resolved by the same catalyst to an enantioenriched diastereomeric product. We recognise that in this first study substrate scope is limited and hope this strategy can be applied to other types of substrates and increase the efficiency of resolution procedures. It is interesting to consider if this kind of selectivity may be operating in any systems of nature and we hope to be able to explore the scope and mechanistic aspects of this reaction.

## Methods

### Synthesis of (1-Azidoethyl)benzene (3)

To a solution of sodium azide (105 mg, 1.61 mmol, 1.10 equiv.) in DMSO (6 mL) was added (1-bromoethyl)benzene (200 μL, 271 mg, 1.47 mmol, 1 equiv.). The reaction mixture was allowed to stir at rt for 2 h. To this mixture was added water (10 mL) and subsequently extracted with ether (3 × 10 mL). The organic extracts were combined, washed with water (2 × 10 mL) and brine (10 mL) and then dried over MgSO_4_, filtered and concentrated under reduced pressure to give (1-azidoethyl)benzene **3** as a pale yellow oil, in 40% (84.0 mg) yield. *Characterisation was in agreement with reported literature values*^40^. ^1^H NMR (300 MHz, CDCl_3_) δ 7.25–7.40 (m, 5H, Ar-*H*), 4.60 (q, *J* 6.8, 1H, C*H*), 1.52 (d, *J* = 6.8, 3H, C*H*_3_); ^13^C NMR (101 MHz, CDCl_3_) δ 140.90, 128.80, 128.15, 126.41, 61.12, 21.59; IR ν_max_ (ATR)/cm^−1^ 3032, 2979, 2090, 1244; MS AP^+^ *m/z* 120.1 [M-N_2_+H]^+^, 105.0 [M-N_3_]^+^; GC (CP-Chirasil-Dex CB), FID, t_(*S*)_ = 28.1 min, t_(*R*)_ = 28.4 min.

### General procedure for the synthesis of enantiopure azides

Under an atmosphere of nitrogen, the corresponding alcohol (2.54 mmol, 1.00 equiv.) was dissolved in anhydrous toluene (4 mL) to this was added diphenylphosphoryl azide (DPPA) (633 μL, 810 mg, 2.94 mmol, 1.20 equiv.). The mixture was cooled to 0 °C for 5 mins and DBU (440 μL, 448 mg, 2.94 mmol, 1.20 equiv.) added. The reaction mixture was allowed to warm to room temperature and stirred for 18 h. The reaction was subsequently quenched with water (10 mL) and aq. HCl 5% v/v (10 mL) and extracted with EtOAc (2 × 10 mL). The combined organic fractions were dried over MgSO_4_ and concentrated under reduced pressure, the crude reside was purified by flash column chromatography (20:1 hexane/EtOAc).

### (*S*)-(1-Azidoethyl)benzene (3 (*S*))

Prepared from (*R*)-phenylethanol according to general procedure, colourless oil **3 (*S*)** (131 mg, 35%). ^1^H NMR (300 MHz, CDCl_3_) δ 7.50–6.97 (m, 5H, Ar-*H*), 4.57 (q, *J* 6.8, 1H, C*H*), 1.49 (d, *J* 6.8, 3H, C*H*_3_); ^13^C NMR (101 MHz, CDCl_3_) δ 140.90, 128.80, 128.14, 126.41, 61.12, 21.80; MS ESI^+^ *m/z* 147.1 [M]^+^, 105.1 [M-N_3_]^+^, 77.0 [M-C_2_H_4_N_3_]^+^; GC (CP-Chirasil-Dex CB), FID, t = 28.1 min.

### (*R*)-(1-Azidoethyl)benzene (3 (*R*))

Prepared from (*S*)-phenylethanol according to the general procedure, colourless oil **3 (*R*)** (150 mg, 40% yield). ^1^H NMR (300 MHz, CDCl_3_) *δ* 7.42–7.18 (m, 5H, Ar-*H*), 4.56 (q, *J* 6.8, 1H, C*H*), 1.48 (d, *J* = 6.8, 3H, C*H*_3_); ^13^C NMR (101 MHz, CDCl_3_) δ 140.90, 128.80, 128.16, 126.41, 61.12, 21.58; MS ESI^+^ *m/z* 147.1 [M]^+^, 105.1 [M-N_3_]^+^, 77.0 [M-C_2_H_4_N_3_]^+^; GC (CP-Chirasil-Dex CB), FID, t = 28.4 min.

### Synthesis of racemic 4-phenyl-1-(1-phenylethyl)-1*H*-1,2,3-triazole (5)

Phenylacetylene (20.0 mg, 0.20 mmol, 1.00 equiv.), (1-azidoethyl)benzene **3** (30.0 mg, 0.20 mmol, 1.00 equiv.) and sodium ascorbate (39.0 mg, 0.20 mmol, 1.00 equiv.) were added to a solution of CuSO_4_||5H_2_O (5.00 mg, 0.020 mmol, 10 mol%) in MeOH (4 mL). The reaction mixture was allowed to stir for 24 h at rt. The reaction was quenched with aq. ammonia solution 5% v/v (5 mL) and extracted with EtOAc (2 × 10 mL). The combined organic extracts were dried over MgSO_4_ and concentrated under reduced pressure to yield 4-phenyl-1-(1-phenylethyl)-1*H*-1,2,3-triazole **5** as a cream solid (13.0 mg, 26%). *Characterisation was consistent with reported literature values*^41^. ^1^H NMR (300 MHz, CDCl_3)_ δ 7.76–7.82 (m, 2H, Ar-*H*), 7.64 (s, 1H, C*H*), 7.26–7.45 (m, 8H, Ar-*H*), 5.86 (q, *J* 7.1, 1H, C*H*,), 2.02 (d, *J* 7.1, 3H, C*H*_3_,); ^13^C NMR (101 MHz, CDCl_3_) δ 147.80, 139.92, 130.67, 129.07, 128.79, 128.58, 128.10, 126.55, 125.69, 118.40, 60.29, 21.32; IR ν_max_ (ATR)/cm^−1^ 3090, 2991; MS ESI^+^ *m/z* 272.1 [M + Na]^+^, 250.1 [M + H]^+^; HPLC (Phenomenex Cellulose 1) acetonitrile/water 60:40, 1.0 mL/min, λ = 210 nm, t = 8.4 and 9.1 min.

### Synthesis of 1-benzyl-3-methyl-3-((1-(1-phenylethyl)-1*H*-1,2,3-triazol-4-yl)methyl)indolin-2-one (6)

To a solution of 1-benzyl-3-methyl-3-(prop-2-yn-1-yl)indolin-2-one **1** (100 mg, 0.36 mmol, 1 equiv.) in acetone (5 mL) was added copper (I) chloride (1.80 mg, 0.018 mmol, 5 mol%), TBTA (9.60 mg, 0.018 mmol, 5 mol%) and (1-azidoethyl)benzene **3** (60.0 mg, 0.40 mmol, 1.10 equiv.) in acetone (1 mL). The mixture was heated to reflux and stirred for 96 h. The reaction mixture was then quenched with aq. ammonia 5% v/v (5 mL) and extracted with EtOAc (3 × 10 mL). The combined organic extracts were washed with water (10 mL), died over MgSO_4_ and concentrated under reduced pressure. The crude residue was purified by automated flash column chromatography Combiflash Rf (0–100% hexane/EtOAc, 12 mins) to yield the triazole **6** as a colourless oil (65.0 mg, 42% yield). Reported as a mixture of diastereoisomers. ^1^H NMR (400 MHz, CDCl_3_) δ 7.32–7.19 (m, 12H (residual solvent ignored), Ar-*H*), 7.15–7.02 (m, 8H, Ar-*H*), 7.00–6.88 (m, 6H, Ar-*H*), 6.72 (s, 1H, Triazole C*H*), 6.68 (s, 1H, Triazole C*H*), 6.56–6.48 (m, 2H, Ar-*H*), 5.54–5.62 (m, 2H, C*H*), 4.55–4.76 (m, 4H, C*H*_2_), 3.16–3.38 (m, 4H, C*H*_2_), 1.79 (d, *J* 7.1, 3H, C*H*_3_), 1.69 (d, *J* 7.1, 3H, C*H*_3_), 1.52 (s, 3H, C*H*_3_), 1.51 (s, 3H, C*H*_3_); ^13^C NMR (101 MHz, CDCl_3_) δ 179.93, 142.97, 142.88, 142.17, 140.24, 140.19, 135.97, 135.86, 132.97, 128.86, 128.71, 128.20, 128.16, 127.80, 127.51, 127.48, 127.29, 127.19, 126.20, 126.17, 123.30, 123.22, 122.52, 122.45, 120.80, 120.39, 108.85, 108.77, 59.78, 59.56, 48.57, 48.54, 43.51, 34.49, 34.45, 23.32, 21.17, 20.81; IR ν_max_ (ATR)/cm^−1^ 3032, 2968, 2925, 1705, 1610; MS ESI^+^ *m/z* 445.2 [M + Na]^+^, 423.2 [M + H]^+^; HRMS ESI^+^ Calculated for C_27_H_26_N_4_ONa^+^ = 445.1999 Found 445.2007; HPLC (Cellulose 3) acetonitrile/water 30:70, 1.0 mL/min, λ = 210 nm, 45 min, 55 min, 59 min, 67 min.

### Synthesis of (*R*)-1-benzyl-3-methyl-3-((1-((*S*)-1-phenylethyl)-1H-1,2,3-triazol-4-yl)methyl)indolin-2-one (6 (*R,S*))

To a solution of (*R*)-1-benzyl-3-methyl-3-(prop-2-yn-1-yl)indolin-2-one **1 (*R*)** (30.0 mg, 0.11 mmol, 1.00 equiv.) in methanol (5 mL) was added (*S*)-(1-azidoethyl)benzene **3 (*S*)** (16.0 mg, 0.11 mmol, 1.00 equiv.), CuSO_4_||5H_2_O (3.00 mg, 0.011 mmol, 10 mol%) and sodium ascorbate (5.00 mg, 0.022 mmol, 20 mol%). The reaction was stirred at 50 °C for 24 h. After this time the reaction was quenched with the addition of aq. ammonia solution 5% v/v (5 mL), the reaction was then extracted with EtOAc (2 × 10 mL). The combined organic fractions were dried over MgSO_4_ and concentrated under reduced pressure. The crude residue was purified by automated flash column chromatography Combiflash Rf (0–100% EtOAc/hexane gradient, 15 mins). This yielded (*R*)-1-benzyl-3-methyl-3-((1-((*S*)-1-phenylethyl)-1H-1,2,3-triazol-4-yl)methyl)indolin-2-one **6 (*R***,***S*)** as a yellow oil (29.0 mg, 62%). ^1^H NMR (400 MHz, CDCl_3_) δ 7.29–7.18 (m, 6H, Ar-*H*), 7.14–7.07 (m, 3H, Ar-*H*), 7.04 (td, *J* 7.7, 1.3, 1H, Ar-*H*), 6.97–6.89 (m, 3H, Ar-*H*), 6.72 (s, 1H, Triazole C*H*), 6.50 (d, *J* 7.7, 1H, Ar-*H*), 5.57 (q, *J* 7.1, 1H, C*H*), 4.65 (ABq, Δδ_AB_ = 0.14, *J* 15.6, 2H, C*H*_2_), 3.27 (ABq, Δδ_AB_ = 0.14, *J* 14.3, 2H, C*H*_2_), 1.79 (d, *J* 7.1, 3H, C*H*_3_), 1.51 (s, 3H, C*H*_3_); ^13^C NMR (101 MHz, CDCl_3_) δ 179.92, 142.88, 142.16, 140.25, 135.91, 132.98, 128.85, 128.70, 128.15, 127.79, 127.48, 127.19, 126.16, 123.22, 122.44, 120.80, 108.84, 59.78, 48.53, 43.50, 34.45, 23.32, 21.16; MS AP^+^ *m/z* 423.2 [M + H]^+^; HRMS AP^+^ Calculated for C_27_H_27_N_4_O^+^ = 423.2179 Found 423.2187; IR ν_max_ (ATR)/cm^−1^ 2925, 2855, 1707, 1489, 1467, 1356, 1174, 855, 741, 698; HPLC (Phenomenex Cellulose 3) acetonitrile/water 30:70, 1.0 mL/min, λ = 250 nm, t = 49 mins.

### Synthesis of 1-benzyl-3-methyl-3-(1-((*S*)*-*1-phenylethyl)-1*H*-1,2,3-triazol-4-yl)indolin-2-one (6 (*rac*-*S*))

Compound **3** (85.0 mg, 0.31 mmol, 1.00 equiv.) and **1 (*S*)** (50.0 mg, 0.34 mmol, 1.10 equiv.) were dissolved in MeOH (5 mL). To this solution was added CuSO_4_.5H_2_O (7.70 mg, 0.031 mmol, 10 mol%) and sodium ascorbate (61.0 mg, 0.31 mmol, 1.00 equiv.) and the resulting mixture stirred for 5 mins at rt. After this time *N*,*N*-diisoproylethylamine (100 μL, 7.40 mg, 5 mol%) was added and the mixture left to stir at rt for 48 h. The reaction mixture was then quenched by the addition of aqueous ammonia solution 5% v/v (5 mL). The resulting solution was extracted with EtOAc (3 × 10 mL). The combined organic fractions were washed with water (10 mL) dried over MgSO_4_ and concentrated under reduced pressure. The recovered crude material was purified by automated flash column chromatography Combiflash Rf (0–100% hexane/EtOAc gradient 12 mins). This yielded 1-benzyl-3-methyl-3-(1-((*S*)*-*1-phenylethyl)-1*H*-1,2,3-triazol-4-yl)indolin-2-one **6 (*rac***,***S*)** as a colourless oil (72.0 mg, 57% yield). The ^1^H NMR spectrum of the product was consistent with compound **6**. HPLC (Phenomenex Cellulose 3) acetonitrile/water 30:70, 1.0 mL/min, λ = 210 nm, 45 min, 67 min.

## Catalysis

### Representative procedure for kinetic resolution of (1-Azidoethyl)benzene (3)

To an oven dried Radley’s multi-reactor tube were added **L1** (6.70 mg, 0.018 mmol, 15.0 mol%), CuCl (1.50 mg, 0.015 mmol, 12.5 mol%) and 2,5-hexanedione (1 mL), these were stirred together at rt for 1 h. To this solution was added phenylacetylene (6.20 mg, 0.06 mmol, 0.50 equiv.) in 2,5-hexanedione (0.5 mL) and the resulting mixture stirred for 15 mins at rt before being cooled to 0 °C and stirred for a further 15 mins. (1-azidoethyl)benzene **3** (17.8 mg, 0.12 mmol, 1.00 equiv.) in 2,5-hexanedione (0.5 mL) was then added and the reaction mixture stirred for 96 h at 0 °C. The reaction mixture was then quenched with the addition of aq. ammonia solution 5% v/v (5 mL) then extracted with ether (2 × 10 mL). The combined organic extracts were dried over MgSO_4_ and concentrated under reduced pressure. Conversion of the reaction was determined through ^1^H NMR spectroscopy of the recovered crude material. Enantiomeric excess was determined by chiral GC. The remaining azide and triazolic product were isolated by automated flash column chromatography Combiflash Rf (0–40% hexane/EtOAc, 15 mins).

### General procedure for the kinetic resolution of 1 with Azide 3

To an oven dried Radley’s multi-reactor tube **L1 (**6.70 mg, 0.018 mmol, 15.0 mol%) and CuCl (1.50 mg, 0.015 mmol, 12.5 mol%) followed by 2,5-hexanedione (1 mL) were added. After stirring at rt for 1 h, compound **1** (33.4 mg, 0.12 mmol, 1 equiv.) dissolved in 2,5-hexanedione (0.5 mL) was added. The reaction mixture was stirred for a further 15 mins before being cooled to 0 °C for 15 min. Azide **3 (*R*)** (8.90 mg, 0.06 mmol, 1 equiv.) dissolved in 2,5-hexanedione (0.5 mL) was then added. The reaction mixture was stirred at 0 °C for 96 h. The reaction was then quenched by addition of aqueous ammonia 5% v/v (5 mL). The reaction mixture was then extracted with ethyl acetate (2 × 10 mL), dried over MgSO_4_ and concentrated under reduced pressure. Conversion was determined by integration of the ^1^H NMR spectrum of the recovered material. The remaining starting material and the triazolic product were subsequently isolated by automated column chromatography Combiflash Rf (0–100% hexane/EtOAc gradient 12 mins). Enantiomeric excess and diastereomer ratio of **6** and enantiomeric excess of **1** were determined by chiral HPLC.

### General procedure for the kinetic resolution of 3 with Alkyne 1

To an oven dried Radley’s multi-reactor tube was added **L1** (6.70 mg, 0.018 mmol, 15.0 mol%) and CuCl (1.50 mg, 0.015 mmol, 12.5 mol%) followed by 2,5-hexanedione (1 mL), the resulting solution was allowed to stir at rt for 1 h. After this time compound **1** (16.7 mg, 0.06 mmol, 0.5 equiv.) dissolved in 2,5-hexanedione (0.5 mL) was added. The reaction mixture was allowed to stir at rt for a further 15 mins after which it was cooled to 0 °C in an ice bath and stirred for a subsequent 15 mins. After this time had passed azide **3** (17.8 mg, 0.12 mmol, 1 equiv.) dissolved in 2,5-hexanedione (0.5 mL) was added. The reaction mixture was stirred for 96 h at 0 °C before being quenched by the addition of aqueous ammonia 5% v/v (5 mL). The resulting solution was extracted with EtOAc (2 × 10 mL), the combined organic fractions were dried over MgSO_4_ and concentrated under reduced pressure. Chiral GC was carried out on the crude recovered material to measure the *ee* of the remaining azide **3**. The remaining crude material was the purified by automated flash column chromatography Combiflash Rf (0–100% hexane/EtOAc gradient, 12 mins). The *dr* and *ee* of the triazolic product was then determined by chiral HPLC.

### General procedure for simultaneous kinetic resolution of 1 and 3

To an oven dried Radley’s multi-reactor tube was added **L1 (**6.70 mg, 0.018 mmol, 15.0 mol%) and CuCl (1.50 mg, 0.015 mmol, 12.5 mol%) followed by 2,5-hexanedione (1 mL), the resulting solution was allowed to stir at rt for 1 h. After this time compound **1** (33.4 mg, 0.12 mmol, 1.00 equiv.) dissolved in 2,5-hexanedione (0.5 mL) was added. The reaction mixture was allowed to stir at rt for a further 15 mins after which it was cooled to 0 °C in an ice bath and stirred for a subsequent 15 mins. After this time had passed azide **3** (17.8 mg, 0.12 mmol, 1 equiv.) dissolved in 2,5-hexanedione (0.5 mL) was added. The reaction mixture was stirred for 96 h at 0 °C before being quenched by the addition of aqueous ammonia 5% v/v (5 mL). The resulting solution was extracted with EtOAc (2 × 10 mL), the combined organic fractions were dried over MgSO_4_ and concentrated under reduced pressure. Chiral GC was carried out on the crude recovered material to measure the *ee* of the remaining azide **3**. The remaining crude material was the purified by automated flash column chromatography Combiflash Rf (0–100% hexane/EtOAc gradient, 12 mins). The *dr* and *ee* of the triazolic product **6** and *ee* of the recovered alkyne **1** was then determined by HPLC with a chiral stationary phase.

Supplementary information is available that includes detailed experimental procedures, NMR spectrums, HPLC traces and X-ray crystallographic information. Citations therein should be referred to in relation to published procedures^40–44^ and a pre-peer reviewed preprint was submitted prior to peer assessment of this manuscript^45^.

## Supplementary Information

Supplementary Information

Supplementary Information

Supplementary Information

Supplementary Information

Supplementary Information
