# Supplementary material for: Coetaneous catalytic kinetic resolution of alkynes and azides through asymmetric triazole formation
Source: Sci Rep. 2019 Oct 21;9:15086. doi: 10.1038/s41598-019-50940-4 (PMC6803658; doi:10.1038/s41598-019-50940-4)
Supplement: Supplementary file 1 — Supplementary Information [file 41598_2019_50940_MOESM1_ESM.pdf]

Supporting Information

Coetaneous catalytic kinetic resolution of alkynes and azides through asymmetric triazole formation

William D. G. Brittain,[a†] Andrew G. Dalling,[a†] Zhenquan Sun,[a,b] Cécile S. Le Duff,[c] Louise Male,[d] Benjamin R. Buckley[e*] and John S. Fossey[a*]

[a] School of Chemistry, University of Birmingham, Edgbaston, Birmingham, West Midlands, B15 2TT, UK
[b] School of Chemistry and Chemical Engineering, Sun Yat-sen University, 135 Xingang Rd. W., Guangzhou 510275, China.
[c] NMR Facility, School of Chemistry, University of Birmingham, Edgbaston, Birmingham, West Midlands, UK
[d] X-Ray Crystallography Facility, School of Chemistry, University of Birmingham, Edgbaston, Birmingham, West Midlands, B15 2TT, UK
[e] Department of Chemistry, Loughborough University, Loughborough, Leicestershire, LE11 3TU, UK

Contents

General **1**
Synthesis **2**
NMR Data **22**
X-Ray Crystallographic Data **32**
References **41**

General

Reagents were used as purchased from suppliers without further purification; in cases where anhydrous solvents were required, they were dried using a solvent purification system (SPS) which is monitored by Karl-Fisher titrations for water levels. ^{1}H NMR spectra were recorded at 300 MHz and 400 MHz using a Bruker AVIII 300 and Bruker AVIII 400 NMR spectrometer and chemical shifts are reported in ppm relative to TMS (δ 0.00). ^{13}C NMR spectra were proton-decoupled and recorded at 101 MHz on a Bruker AVIII 400 NMR spectrometer, with chemical shifts reported in ppm relative to chloroform-d (δ 77.36). Coupling constants (*J*) are measured in Hertz. 2D NMR techniques were used when required to confirm compound identity. IR spectra were recorded on a PerkinElmer 100FT-IR spectrometer at room temperature using ATR. HPLC analysis was performed using a Shimadzu LC2010 and Agilent 1260 Infinity with Phenomenex Lux cellulose 1 and 3 chiral columns where specified, chromatograms were recorded at 210, 220, 254 and 280 nm. GC analysis was carried out using a Varian 430-GC with FID and CP-Chirasil-Dex CB column with oven conditions of 60-150 °C in 60 min. Column chromatography was carried out using a Combiflash R*f* 200i and column traces were recorded at two UV wavelengths (254 nm and 280 nm).

Synthesis

Synthesis of (1-Azidoethyl)benzene (3)

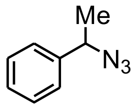

3

To a solution of sodium azide (105 mg, 1.61 mmol, 1.10 equiv.) in DMSO (6 mL) was added (1-bromoethyl)benzene (200 μL, 271 mg, 1.47 mmol, 1 equiv.). The reaction mixture was allowed to stir at rt for 2 h. To this mixture was added water (10 mL) and subsequently extracted with ether (3 x 10 mL). The organic extracts were combined, washed with water (2 x 10 mL) and brine (10 mL) and then dried over $MgSO_4$, filtered and concentrated under reduced pressure to give (1-azidoethyl)benzene **3** as a pale yellow oil, in 40% (84.0 mg) yield. *Characterisation was in agreement with reported literature values.*[1] ^{1}H NMR (300 MHz, $CDCl_3$) δ 7.25 –7.40 (m, 5H, Ar-*H*), 4.60 (q, *J* = 6.8, 1H, C*H*), 1.52 (d, *J* = 6.8, 3H, CH_3); ^{13}C NMR (101 MHz, $CDCl_3$) δ 140.90, 128.80, 128.15, 126.41, 61.12, 21.59; IR ν_{max} (ATR)/cm^{-1} 3032, 2979, 2090, 1244; MS AP$^+$ *m/z* 120.1 [M-N_2+H]$^+$, 105.0 [M-N_3]$^+$; GC (CP-Chirasil-Dex CB), FID, $t_{(S)}$ = 28.1 min, $t_{(R)}$ = 28.4 min.

General Procedure for the Synthesis of Enantiopure Azides

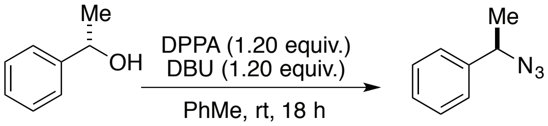

Under an atmosphere of nitrogen, the corresponding alcohol (2.54 mmol, 1.00 equiv.) was dissolved in anhydrous toluene (4 mL) to this was added diphenylphosphoryl azide (DPPA) (633 μL, 810 mg, 2.94 mmol, 1.20 equiv.). The mixture was cooled to 0 °C for 5 mins and DBU (440 μL, 448 mg, 2.94 mmol, 1.20 equiv.) added. The reaction mixture was allowed to warm to room temperature and stirred for 18 h. The reaction was subsequently quenched with water (10 mL) and aq. HCl 5% v/v (10 mL) and extracted with EtOAc (2 x 10 mL). The combined organic fractions were dried over $MgSO_4$ and concentrated under reduced pressure, the crude reside was purified by flash column chromatography (20:1 hexane/EtOAc).

(*S*)-(1-Azidoethyl)benzene (3 (*S*))

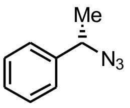

3 (S)

Prepared from (*R*)-phenylethanol according to general procedure, colourless oil **3** (***S***) (131 mg, 35%). ^{1}H NMR (300 MHz, $CDCl_3$) δ 7.50 – 6.97 (m, 5H, Ar-*H*), 4.57 (q, *J* = 6.8, 1H, C*H*), 1.49

(d, J = 6.8, 3H, CH_3); ^{13}C NMR (101 MHz, $CDCl_3$) δ 140.90, 128.80, 128.14, 126.41, 61.12, 21.80; MS ESI$^+$ m/z 147.1 [M]$^+$, 105.1 [M-N_3]$^+$, 77.0 [M-$C_2H_4N_3$]$^+$; GC (CP-Chirasil-Dex CB), FID, t = 28.1 min.

(*R*)-(1-Azidoethyl)benzene (3 (*R*))

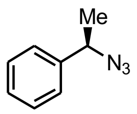

3 (R)

Prepared from (*S*)-phenylethanol according to the general procedure, colourless oil **3 (*R*)** (150 mg, 40% yield). ^{1}H NMR (300 MHz, $CDCl_3$) δ 7.42 – 7.18 (m, 5H, Ar-H), 4.56 (q, J = 6.8, 1H, CH), 1.48 (d, J = 6.8, 3H, CH_3); ^{13}C NMR (101 MHz, $CDCl_3$) δ 140.90, 128.80, 128.16, 126.41, 61.12, 21.58; MS ESI$^+$ m/z 147.1 [M]$^+$, 105.1 [M-N_3]$^+$, 77.0 [M-$C_2H_4N_3$]$^+$; GC (CP-Chirasil-Dex CB), FID, t = 28.4 min.

Synthesis of Racemic 4-phenyl-1-(1-phenylethyl)-1*H*-1,2,3-triazole (5)

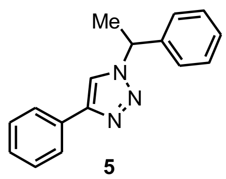

5

Phenylacetylene (20.0 mg, 0.20 mmol, 1.00 equiv.), (1-azidoethyl)benzene **3** (30.0 mg, 0.20 mmol, 1.00 equiv.) and sodium ascorbate (39.0 mg, 0.20 mmol, 1.00 equiv.) were added to a solution of $CuSO_4 \cdot 5H_2O$ (5.00 mg, 0.020 mmol, 10 mol%) in MeOH (4 mL). The reaction mixture was allowed to stir for 24 h at rt. The reaction was quenched with aq. ammonia solution 5% v/v (5 mL) and extracted with EtOAc (2 x 10 mL). The combined organic extracts were dried over $MgSO_4$ and concentrated under reduced pressure to yield 4-phenyl-1-(1-phenylethyl)-1*H*-1,2,3-triazole **5** as a cream solid (13.0 mg, 26%). *Characterisation was consistent with reported literature values.*[2] ^{1}H NMR (300 MHz, $CDCl_3$) δ 7.76 – 7.82 (m, 2H, Ar-H), 7.64 (s, 1H, CH), 7.26 – 7.45 (m, 8H, Ar-H), 5.86 (q, J = 7.1, 1H, CH,), 2.02 (d, J = 7.1, 3H, CH_3,); ^{13}C NMR (101 MHz, $CDCl_3$) δ 147.80, 139.92, 130.67, 129.07, 128.79, 128.58, 128.10, 126.55, 125.69, 118.40, 60.29, 21.32; IR ν_{max} (ATR)/cm^{-1} 3090, 2991; MS ESI$^+$ m/z 272.1 [M+Na]$^+$, 250.1 [M+H]$^+$; HPLC (Phenomenex Cellulose 1) acetonitrile/water 60:40, 1.0 mL/min, λ = 210 nm, t = 8.4 and 9.1 min.

Synthesis of 1-benzyl-3-methyl-3-((1-(1-phenylethyl)-1*H*-1,2,3-triazol-4-yl)methyl)indolin-2-one (6)

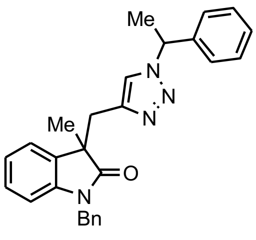

6

To a solution of 1-benzyl-3-methyl-3-(prop-2-yn-1-yl)indolin-2-one **1** (100 mg, 0.36 mmol, 1 equiv.) in acetone (5 mL) was added copper (I) chloride (1.80 mg, 0.018 mmol, 5 mol%), TBTA (9.60 mg, 0.018 mmol, 5 mol%) and (1-azidoethyl)benzene **3**

(60.0 mg, 0.40 mmol, 1.10 equiv.) in acetone (1 mL). The mixture was heated to reflux and stirred for 96 h. The reaction mixture was then quenched with aq. ammonia 5% v/v (5 mL) and extracted with EtOAc (3 x 10 mL). The combined organic extracts were washed with water (10 mL), died over $MgSO_4$ and concentrated under reduced pressure. The crude residue was purified by automated flash column chromatography Combiflash Rf (0-100% hexane/EtOAc, 12 mins) to yield the triazole **6** as a colourless oil (65.0 mg, 42% yield). Reported as a mixture of diastereoisomers. ^{1}H NMR (400 MHz, $CDCl_3$) δ 7.32-7.19 (m, 12H (residual solvent ignored), Ar-*H*), 7.15-7.02 (m, 8H, Ar-*H*), 7.00-6.88 (m, 6H, Ar-*H*), 6.72 (s, 1H, Triazole C*H*), 6.68 (s, 1H, Triazole C*H*), 6.56-6.48 (m, 2H, Ar-*H*), 5.54-5.62 (m, 2H, C*H*), 4.55-4.76 (m, 4H, CH_2), 3.16-3.38 (m, 4H, CH_2), 1.79 (d, J = 7.1, 3H, CH_3), 1.69 (d, J = 7.1, 3H, CH_3), 1.52 (s, 3H, CH_3), 1.51 (s, 3H, CH_3); ^{13}C NMR (101 MHz, $CDCl_3$) δ 179.93, 142.97, 142.88, 142.17, 140.24, 140.19, 135.97, 135.86, 132.97, 128.86, 128.71, 128.20, 128.16, 127.80, 127.51, 127.48, 127.29, 127.19, 126.20, 126.17, 123.30, 123.22, 122.52, 122.45, 120.80, 120.39, 108.85, 108.77, 59.78, 59.56, 48.57, 48.54, 43.51, 34.49, 34.45, 23.32, 21.17, 20.81; IR ν_{max} (ATR)/cm^{-1} 3032, 2968, 2925, 1705, 1610; MS ESI$^+$ *m/z* 445.2 [M+Na]$^+$, 423.2 [M+H]$^+$; HRMS ESI$^+$ Calculated for $C_{27}H_{26}N_4ONa^+$ = 445.1999 Found 445.2007; HPLC (Cellulose 3) acetonitrile/water 30:70, 1.0 mL/min, λ = 210 nm, 45 min, 55 min, 59 min, 67 min.

Synthesis of (*R*)-1-benzyl-3-methyl-3-((1-((*S*)-1-phenylethyl)-1*H*-1,2,3-triazol-4-yl)methyl)indolin-2-one (6 (*R,S*))

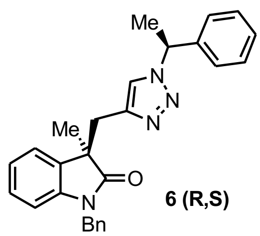

6 (R,S)

To a solution of (*R*)-1-benzyl-3-methyl-3-(prop-2-yn-1-yl)indolin-2-one **1** (***R***) (30.0 mg, 0.11 mmol, 1.00 equiv.) in methanol (5 mL) was added (*S*)-(1-azidoethyl)benzene **3** (***S***) (16.0 mg, 0.11 mmol, 1.00 equiv.), $CuSO_4 \cdot 5H_2O$ (3.00 mg, 0.011 mmol, 10 mol%) and sodium ascorbate (5.00 mg, 0.022 mmol, 20 mol%). The reaction was stirred at 50 °C for 24 h. After this time the reaction was quenched with the addition of aq. ammonia solution 5% v/v (5 mL), the reaction was then extracted with EtOAc (2 x 10 mL). The combined organic fractions were dried over $MgSO_4$ and concentrated under reduced pressure. The crude residue was purified by automated flash column chromatography Combiflash Rf (0-100% EtOAc/hexane gradient, 15 mins). This yielded (*R*)-1-benzyl-3-methyl-3-((1-((*S*)-1-phenylethyl)-1H-1,2,3-triazol-4-yl)methyl)indolin-2-one **6** (***R,S***) as a yellow oil (29.0 mg, 62%). ^{1}H NMR (400 MHz, $CDCl_3$) δ 7.29 – 7.18 (m, 6H, Ar-*H*), 7.14 –

7.07 (m, 3H, Ar-*H*), 7.04 (td, *J* = 7.7, 1.3, 1H, Ar-*H*), 6.97 – 6.89 (m, 3H, Ar-*H*), 6.72 (s, 1H, Triazole C*H*), 6.50 (d, *J* = 7.7, 1H, Ar-*H*), 5.57 (q, *J* = 7.1, 1H, C*H*), 4.65 (ABq, $\Delta\delta_{AB}$ = 0.14, *J* = 15.6, 2H, C*H*$_2$), 3.27 (ABq, $\Delta\delta_{AB}$ = 0.14, *J* = 14.3, 2H, C*H*$_2$), 1.79 (d, *J* = 7.1, 3H, C*H*$_3$), 1.51 (s, 3H, C*H*$_3$); ^{13}C NMR (101 MHz, $CDCl_3$) δ 179.92, 142.88, 142.16, 140.25, 135.91, 132.98, 128.85, 128.70, 128.15, 127.79, 127.48, 127.19, 126.16, 123.22, 122.44, 120.80, 108.84, 59.78, 48.53, 43.50, 34.45, 23.32, 21.16; MS AP$^+$ *m/z* 423.2 [M+H]$^+$; HRMS AP$^+$ Calculated for $C_{27}H_{27}N_4O^+$ = 423.2179 Found 423.2187; IR ν_{max} (ATR)/cm^{-1} 2925, 2855, 1707, 1489, 1467, 1356, 1174, 855, 741, 698; HPLC (Phenomenex Cellulose 3) acetonitrile/water 30:70, 1.0 mL/min, λ = 250 nm, t = 49 mins.

Synthesis of 1-benzyl-3-(3-iodoprop-2-yn-1-yl)-3-methylindolin-2-one (7)

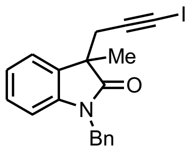

7

Iodine (51.0 mg, 0.20 mmol) was dissolved in toluene (5 mL) and morpholine (271 mg, 3.00 mmol) was added. The mixture was stirred at room temperature for 30 min. After that, the racemic 1-benzyl-3-methyl-3-(prop-2-yn-1-yl)indolin-2-one **1** (50.0 mg, 0.18 mmol) was added, and the mixture was stirred at 50 °C for 72 h. Water (5 mL) was then added and subsequently extracted with ethyl acetate (2 x 5 mL). The combined organic layers were dried over $MgSO_4$. The solvent was removed under reduced pressure. The crude material was then purified by automated column chromatography Combiflash Rf: 0-100% hexane/EtOAc gradient, 20 mins). To yield 1-benzyl-3-(3-iodoprop-2-yn-1-yl)-3-methylindolin-2-one **7** as a pale yellow solid (66.0 mg, 92%).

^{1}H NMR (400 MHz, $CDCl_3$) δ 7.39 – 7.22 (m, 6H, Ar-*H*), 7.17 (td, *J* = 7.7, 1.3, 1H, Ar-*H*), 7.05 (td, *J* = 7.5, 1.1, 1H, Ar-*H*), 6.71 (d, *J* = 7.7, 1H, Ar-*H*), 4.92 (ABq, $\Delta\delta_{AB}$ = 0.24, *J* = 15.8, 2H, C*H*$_2$), 2.85 (ABq, $\Delta\delta_{AB}$ = 0.16, *J* = 16.6, 2H, C*H*$_2$), 1.49 (s, 3H, C*H*$_3$); ^{13}C NMR (101 MHz, $CDCl_3$) δ 179.24, 142.07, 135.74, 132.77, 128.90, 128.19, 127.51, 127.15, 123.22, 122.68, 109.19, 90.04, 47.13, 43.80, 29.99, 22.37, -3.61; IR ν_{max} (ATR)/cm^{-1} 2964, 2924, 1714, 1610, 1490, 1455, 1359, 1321, 1182, 1154, 754; MS ESI$^+$ *m/z* 424.0 [M+Na]$^+$; HRMS ESI$^+$ Calculated for $C_{19}H_{16}NONaI^+$ = 424.0168 Found 424.0178; MP 140 – 143°C; HPLC (Phenomenex Cellulose 3) acetonitrile/water 50:50, 1.0 mL/min, λ = 210 nm, $t_{(S)}$ = 10.7 min, $t_{(R)}$ = 14.4 min.

Synthesis of (*R*)-1-benzyl-3-(3-iodoprop-2-yn-1-yl)-3-methylindolin-2-one (7 (*R*))

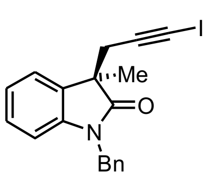

7 (R)

Iodine (51.0 mg, 0.20 mmol) was dissolved in toluene (2 mL) and morpholine (271 mg, 3.0 mmol) was added. The mixture was stirred at room temperature for 30 min. After that, solution of (*R*)-1-benzyl-3-methyl-3-(prop-2-yn-1-yl)indolin-2-one **1** (***R***) (50 mg, 0.18 mmol) in toluene (3 ml) was added, and the mixture was stirred at 50 °C for 72 h. Water (5 mL) was then added and subsequently extracted with ethyl acetate (2 x 5 mL). The combined organic layers were dried over $MgSO_4$, and the solvent was removed under reduced pressure. The crude material was then purified by automated column chromatography Combiflash Rf (0-100% hexane/EtOAc gradient, 20 mins). To yield (*R*)-1-benzyl-3-(3-iodoprop-2-yn-1-yl)-3-methylindolin-2-one **7** (***R***) pale yellow solid (54.0 mg, 74%). ^{1}H NMR (400 MHz, $CDCl_3$) δ 7.38 – 7.22 (m, 6H, Ar-*H*), 7.17 (td, *J* = 7.7, 1.3, 1H, Ar-*H*), 7.05 (td, *J* = 7.5, 1.0, 1H, Ar-*H*), 6.71 (d, *J* = 7.7, 1H, Ar-*H*), 4.92 (ABq, $\Delta\delta_{AB}$ = 0.23, *J* = 15.7, 2H, CH_2), 2.85 (ABq, $\Delta\delta_{AB}$ = 0.16, *J* = 16.6, 2H, CH_2), 1.49 (s, 3H, CH_3); ^{13}C NMR (101 MHz, $CDCl_3$) δ 179.24, 142.07, 135.74, 132.77, 128.90, 128.19, 127.51, 127.15, 123.22, 122.68, 109.20, 90.04, 47.13, 43.80, 29.99, 22.37, -3.61; IR ν_{max} (ATR)/cm^{-1} 2984, 2924, 1682, 1609, 1490, 1467, 1451, 1435, 1422, 1383, 1365, 1354, 1321, 1180, 1076, 732; MS ES$^+$ *m/z* 424.0 [M+Na]$^+$; HRMS ESI$^+$ Calculated for $C_{19}H_{16}NONaI^+$ = 424.0168 Found 424.0168; MP 158 – 159°C; HPLC (Phenomenex Cellulose 3) acetonitrile/water 50:50, 1.0 mL/min, λ = 210 nm, t_R = 14.7 min.

To quantify which peaks in the HPLC traces were due to the diasteroisomer pairs and which were enantiomer pairs a series of resolutions were carried out using enantiopure starting alkyne or azide. This in combination with an enantiopure crystal structure of iodo substituted alkyne **7** we could confirm the absolute configurations (Figure 1). From these resolutions, it was possible to determine the stereochemistry of the major diastereoisomer as (*R,S*) and the recovered alkyne and azide as (*S*) and (*R*) respectively.

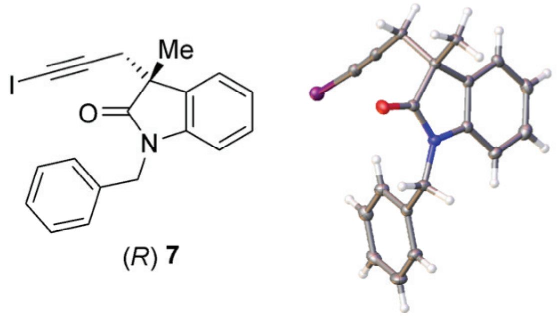

Figure 1. *Crystal Structure of Single Enantiomer of Iodo Alkyne 7. Stereocentre was Determined as (R) by Anomalous Dispersion. Further evidenced by comparison of space group to a racemic crystal of 7, P 1 21 1 (single enantiomer) vs P 1 21/c 1 (see below).*

Synthesis of 1-benzyl-3-methyl-3-(1-((*S*)-1-phenylethyl)-1*H*-1,2,3-triazol-4-yl)indolin-2-one (6 (*rac*-*S*))

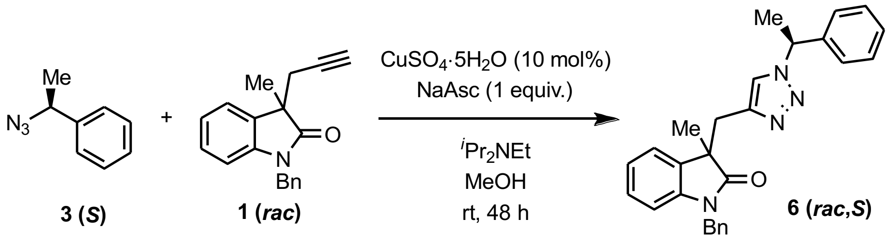

Compound **3** (85.0 mg, 0.31 mmol, 1.00 equiv.) and **1 (*S*)** (50.0 mg, 0.34 mmol, 1.10 equiv.) were dissolved in MeOH (5 mL). To this solution was added $CuSO_4.5H_2O$ (7.70 mg, 0.031 mmol, 10 mol%) and sodium ascorbate (61.0 mg, 0.31 mmol, 1.00 equiv.) and the resulting mixture stirred for 5 mins at rt. After this time *N*,*N*-diisoproylethylamine (100 µL, 7.40 mg, 5 mol%) was added and the mixture left to stir at rt for 48 h. The reaction mixture was then quenched by the addition of aqueous ammonia solution 5% v/v (5 mL). The resulting solution was extracted with EtOAc (3 x 10 mL). The combined organic fractions were washed with water (10 mL) dried over $MgSO_4$ and concentrated under reduced pressure. The recovered crude material was purified by automated flash column chromatography Combiflash Rf (0-100% hexane/EtOAc gradient 12 mins). This yielded 1-benzyl-3-methyl-3-(1-((*S*)-1-phenylethyl)-1*H*-1,2,3-triazol-4-yl)indolin-2-one **6 (*rac*,*S*)** as a colourless oil (72.0 mg, 57% yield). The ^{1}H NMR spectrum of the product was consistent with compound **6**. HPLC (Phenomenex Cellulose 3) acetonitrile/water 30:70, 1.0 mL/min, λ = 210 nm, 45 min, 67 min.

Catalysis

Representative Procedure for Kinetic Resolution of (1-Azidoethyl)benzene (3)

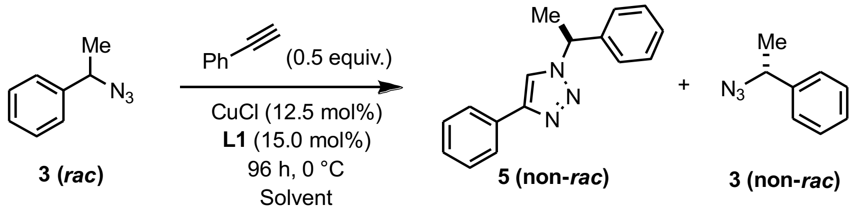

To an oven dried Radley's multi-reactor tube were added **L1** (6.70 mg, 0.018 mmol, 15.0 mol%), CuCl (1.50 mg, 0.015 mmol, 12.5 mol%) and 2,5-hexanedione (1 mL), these were stirred together at rt for 1 h. To this solution was added phenylacetylene (6.20 mg, 0.06 mmol, 0.50 equiv.) in 2,5-hexanedione (0.5 mL) and the resulting mixture stirred for 15 mins at rt before being cooled to 0 °C and stirred for a further 15 mins. (1-azidoethyl)benzene **3** (17.8 mg, 0.12 mmol, 1.00 equiv.) in 2,5-hexanedione (0.5 mL) was then added and the reaction mixture stirred for 96 h at 0 °C. The reaction mixture was then quenched with the addition of aq. ammonia solution 5% v/v (5 mL) then extracted with ether (2 x 10 mL). The combined organic extracts were dried over $MgSO_4$ and concentrated under reduced pressure. Conversion of the reaction was determined through ^1H NMR spectroscopy of the recovered crude material. Enantiomeric excess was determined by chiral GC. The remaining azide and triazolic product were isolated by automated flash column chromatography Combiflash Rf (0-40% hexane/EtOAc, 15 mins).

General Procedure for the Kinetic Resolution of 1 with Azide 3

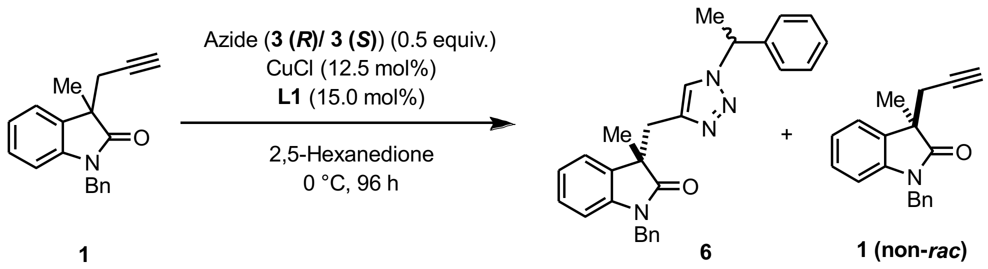

To an oven dried Radley's multi-reactor tube **L1** (6.70 mg, 0.018 mmol, 15.0 mol%) and CuCl (1.50 mg, 0.015 mmol, 12.5 mol%) followed by 2,5-hexanedione (1 mL) were added. After stirring at rt for 1 h, compound **1** (33.4 mg, 0.12 mmol, 1 equiv.) dissolved in 2,5-hexanedione (0.5 mL) was added. The reaction mixture was stirred for a further 15 mins before being cooled to 0 °C for 15 min. Azide **3** (***R***) (8.90 mg, 0.06 mmol, 1 equiv.) dissolved in 2,5-hexanedione (0.5 mL) was then added. The reaction mixture was stirred at 0 °C for 96 h. The reaction was then quenched by addition of aqueous ammonia 5% v/v (5 mL). The reaction mixture was then extracted with ethyl acetate (2 x 10 mL), dried over $MgSO_4$ and concentrated under

reduced pressure. Conversion was determined by integration of the ^{1}H NMR spectrum of the recovered material. The remaining starting material and the triazolic product were subsequently isolated by automated column chromatography Combiflash Rf (0-100% hexane/EtOAc gradient 12 mins). Enantiomeric excess and diastereomer ratio of **6** and enantiomeric excess of **1** were determined by chiral HPLC.

General Procedure for the Kinetic Resolution of 3 with Alkyne 1

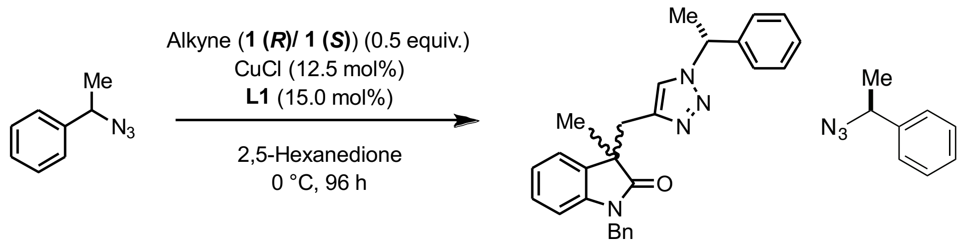

3 (*rac*) **6** + **3 (non-*rac*)**

To an oven dried Radley's multi-reactor tube was added **L1** (6.70 mg, 0.018 mmol, 15.0 mol%) and CuCl (1.50 mg, 0.015 mmol, 12.5 mol%) followed by 2,5-hexanedione (1 mL), the resulting solution was allowed to stir at rt for 1 h. After this time compound **1** (16.7 mg, 0.06 mmol, 0.5 equiv.) dissolved in 2,5-hexanedione (0.5 mL) was added. The reaction mixture was allowed to stir at rt for a further 15 mins after which it was cooled to 0 °C in an ice bath and stirred for a subsequent 15 mins. After this time had passed azide **3** (17.8 mg, 0.12 mmol, 1 equiv.) dissolved in 2,5-hexanedione (0.5 mL) was added. The reaction mixture was stirred for 96 h at 0 °C before being quenched by the addition of aqueous ammonia 5% v/v (5 mL). The resulting solution was extracted with EtOAc (2 x 10 mL), the combined organic fractions were dried over $MgSO_4$ and concentrated under reduced pressure. Chiral GC was carried out on the crude recovered material to measure the *ee* of the remaining azide **3**. The remaining crude material was the purified by automated flash column chromatography Combiflash Rf (0-100% hexane/EtOAc gradient, 12 mins). The *dr* and *ee* of the triazolic product was then determined by chiral HPLC.

General Procedure for Simultaneous Kinetic Resolution of 1 and 3

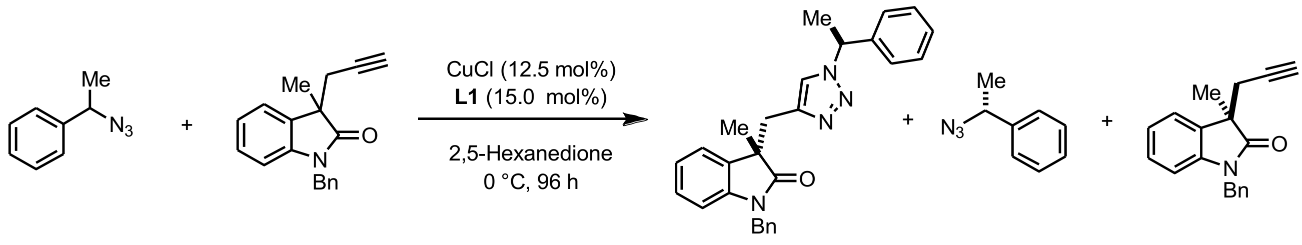

3 (*rac*) **1 (*rac*)** **6** **3 (non-*rac*)** **1 (non-*rac*)**

To an oven dried Radley's multi-reactor tube was added **L1** (6.70 mg, 0.018 mmol, 15.0 mol%) and CuCl (1.50 mg, 0.015 mmol, 12.5 mol%) followed by 2,5-hexanedione (1 mL), the resulting solution was allowed to stir at rt for 1 h. After this time compound **1** (33.4 mg, 0.12 mmol, 1.00 equiv.) dissolved in 2,5-hexanedione (0.5 mL) was added. The reaction mixture was allowed to stir at rt for a further 15 mins after which it was cooled to 0 °C in an ice bath and stirred for a subsequent 15 mins. After this time had passed azide **3** (17.8 mg, 0.12 mmol, 1 equiv.) dissolved in 2,5-hexanedione (0.5 mL) was added. The reaction mixture was stirred for 96 h at 0 °C before being quenched by the addition of aqueous ammonia 5% v/v (5 mL). The resulting solution was extracted with EtOAc (2 x 10 mL), the combined organic fractions were dried over $MgSO_4$ and concentrated under reduced pressure. Chiral GC was carried out on the crude recovered material to measure the *ee* of the remaining azide **3**. The remaining crude material was the purified by automated flash column chromatography Combiflash Rf (0-100% hexane/EtOAc gradient, 12 mins). The *dr* and *ee* of the triazolic product **6** and *ee* of the recovered alkyne **1** was then determined by chiral HPLC.

^{1}H NMR Spectroscopy Conversion Data

Representative Determination of Conversion of Alkyne to Triazole *via* ^{1}H NMR Spectroscopy

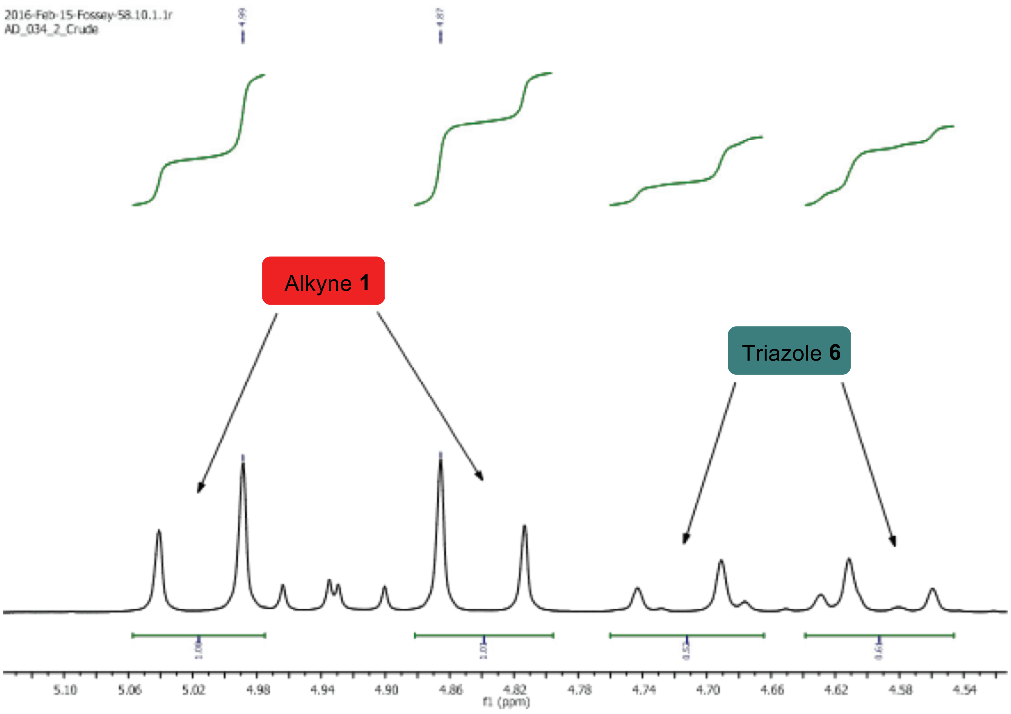

Figure 2 *^{1}H NMR spectrum of crude kinetic resolution mixture showing comparison of signals related to starting material and product allowing calculation of conversion.*

Conversion of alkyne **1** to triazole **6** was determined by comparison of the integrations of signals in the crude ^{1}H NMR spectrum of the reaction mixture at the end of the kinetic resolution. The AB system of compound **1** was compared with the AB system centred at 4.65 ppm of compound **6**. A representative example is shown above.

Integration per proton in **1** = 1.005

Integration per proton in **6** = $\frac{0.52+0.61}{2} = 0.565$

Conversion = $\frac{0.565}{1.005+0.565} \times 100 = 36\%$

Unlocked ^{1}H NMR Study of the Kinetic Resolution of 3 in 2,5-Hexanedione

NMR experiments were run on a Bruker AVANCE spectrometer operating at 400 MHz for ^{1}H and equipped with a 5 mm Broadband (BBO) z-gradient probe.

Samples in non-deuterated 2,5-hexanedione were run unlocked and shimmed directly on the solvent peaks. The ^{1}H spectral width was set to 16 ppm with 32K data points. Measurements were acquired with 128 scans, a delay between scans of 10 s (to allow sufficient time for spin relaxation and therefore accurate integration), of 32k data points and an acquisition time of 2.5 s.

The spectra were indirectly calibrated to the residual ^{1}H peak of deuterated chloroform at 7.26 ppm. Data were processed using an exponential multiplication window function with a line broadening of 2 Hz. The final spectral resolution was 0.19 Hz per point.
