# Supplementary material for: Coetaneous catalytic kinetic resolution of alkynes and azides through asymmetric triazole formation
Source: Sci Rep. 2019 Oct 21;9:15086. doi: 10.1038/s41598-019-50940-4 (PMC6803658; doi:10.1038/s41598-019-50940-4)

# checkCIF/PLATON report

Structure factors have been supplied for datablock(s) ws09_iodide_gaussian

THIS REPORT IS FOR GUIDANCE ONLY. IF USED AS PART OF A REVIEW PROCEDURE FOR PUBLICATION, IT SHOULD NOT REPLACE THE EXPERTISE OF AN EXPERIENCED CRYSTALLOGRAPHIC REFEREE.

No syntax errors found. CIF dictionary Interpreting this report

## Datablock: ws09_iodide_gaussian

---

```
Bond precision:  C-C = 0.0032 A         Wavelength=1.54184

Cell:          a=15.31276(18)   b=8.17349(9)         c=15.58899(17)
               alpha=90         beta=106.1831(12)    gamma=90
Temperature:   100 K

                Calculated                 Reported
Volume          1873.79(4)                 1873.79(4)
Space group     P 21/c                     P 1 21/c 1
Hall group      -P 2ybc                    -P 2ybc
Moiety formula  C19 H16 I N O, C2 H3 N     C19 H16 I N O, C2 H3 N
Sum formula     C21 H19 I N2 O             C21 H19 I N2 O
Mr              442.28                     442.28
Dx,g cm-3       1.568                      1.568
Z               4                          4
Mu (mm-1)       13.502                     13.502
F000            880.0                      880.0
F000'           881.01
h,k,lmax        18,9,19                    18,9,19
Nref            3556                       3548
Tmin,Tmax       0.216,0.384                0.696,0.845
Tmin'           0.096

Correction method= # Reported T Limits: Tmin=0.696 Tmax=0.845
AbsCorr = GAUSSIAN

Data completeness= 0.998          Theta(max)= 70.057

R(reflections)= 0.0211( 3367)     wR2(reflections)= 0.0544( 3548)

S = 1.077                  Npar= 228
```

---

The following ALERTS were generated. Each ALERT has the format
**test-name_ALERT_alert-type_alert-level**.
Click on the hyperlinks for more details of the test.

### Alert level C

```
PLAT911_ALERT_3_C Missing # FCF Refl Between THmin & STh/L=  0.600          3 Report
```

### Alert level G

```
PLAT431_ALERT_2_G Short Inter HL..A  Contact I1     ..  N101    ..       2.91 Ang.
PLAT793_ALERT_4_G The Model has Chirality at C2      (Centro SPGR)          R Verify
PLAT912_ALERT_4_G Missing # of FCF Reflections Above STh/L=  0.600          5 Note
PLAT933_ALERT_2_G Number of OMIT Records in Embedded .res File ...          1 Note
PLAT955_ALERT_1_G Reported (CIF) and Actual (FCF) Lmax Differ by .          1 Units
PLAT978_ALERT_2_G Number C-C Bonds with Positive Residual Density.         11 Note
```

---

```
   0 ALERT level A = Most likely a serious problem - resolve or explain
   0 ALERT level B = A potentially serious problem, consider carefully
   1 ALERT level C = Check. Ensure it is not caused by an omission or oversight
   6 ALERT level G = General information/check it is not something unexpected

   1 ALERT type 1 CIF construction/syntax error, inconsistent or missing data
   3 ALERT type 2 Indicator that the structure model may be wrong or deficient
   1 ALERT type 3 Indicator that the structure quality may be low
   2 ALERT type 4 Improvement, methodology, query or suggestion
   0 ALERT type 5 Informative message, check
```

---

It is advisable to attempt to resolve as many as possible of the alerts in all categories. Often the minor alerts point to easily fixed oversights, errors and omissions in your CIF or refinement strategy, so attention to these fine details can be worthwhile. In order to resolve some of the more serious problems it may be necessary to carry out additional measurements or structure refinements. However, the purpose of your study may justify the reported deviations and the more serious of these should normally be commented upon in the discussion or experimental section of a paper or in the "special_details" fields of the CIF. checkCIF was carefully designed to identify outliers and unusual parameters, but every test has its limitations and alerts that are not important in a particular case may appear. Conversely, the absence of alerts does not guarantee there are no aspects of the results needing attention. It is up to the individual to critically assess their own results and, if necessary, seek expert advice.

**Publication of your CIF in IUCr journals**

A basic structural check has been run on your CIF. These basic checks will be run on all CIFs submitted for publication in IUCr journals (*Acta Crystallographica*, *Journal of Applied Crystallography*, *Journal of Synchrotron Radiation*); however, if you intend to submit to *Acta Crystallographica Section C* or *E* or *IUCrData*, you should make sure that full publication checks are run on the final version of your CIF prior to submission.

**Publication of your CIF in other journals**

Please refer to the *Notes for Authors* of the relevant journal for any special instructions relating to CIF submission.

---

**PLATON version of 24/11/2016; check.def file version of 23/11/2016**

**Datablock ws09_iodide_gaussian** - ellipsoid plot

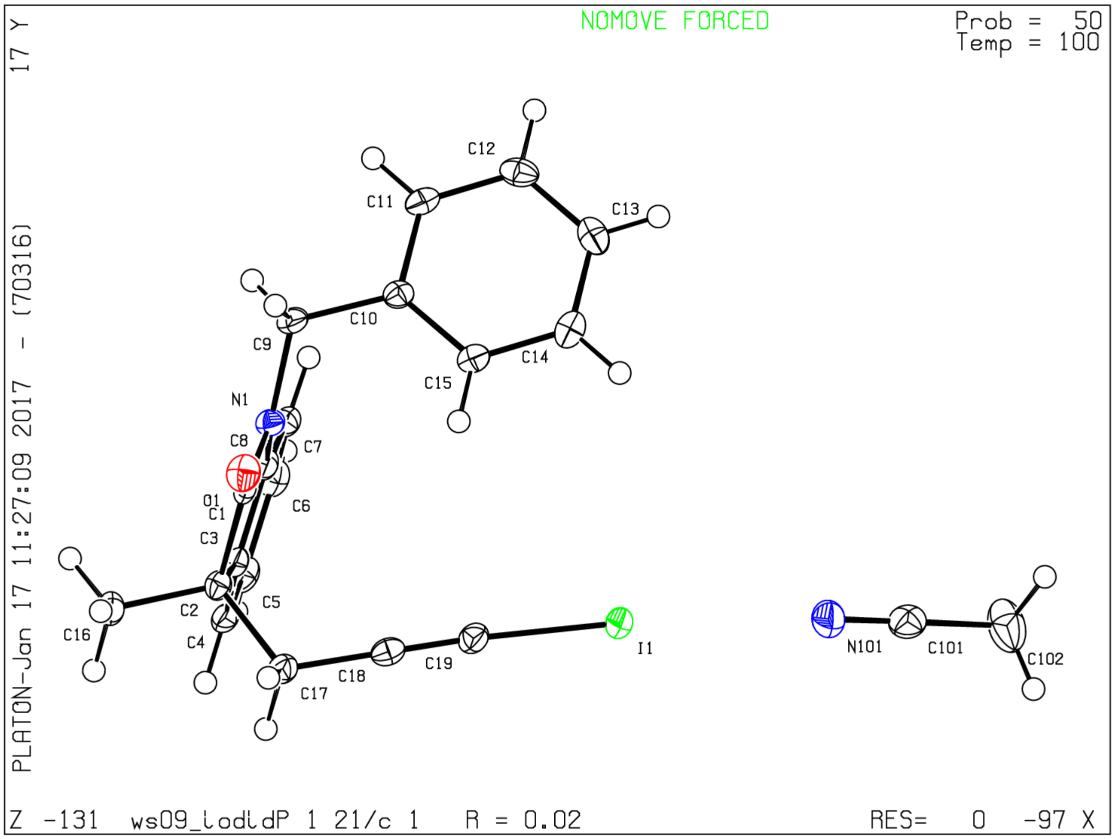

Supplement: Supplementary file 3 — Supplementary Information [file 41598_2019_50940_MOESM3_ESM.pdf]
