# Supplementary material for: Coetaneous catalytic kinetic resolution of alkynes and azides through asymmetric triazole formation
Source: Sci Rep. 2019 Oct 21;9:15086. doi: 10.1038/s41598-019-50940-4 (PMC6803658; doi:10.1038/s41598-019-50940-4)

checkCIF/PLATON report

Structure factors have been supplied for datablock(s) ws09a1_gaussian

THIS REPORT IS FOR GUIDANCE ONLY. IF USED AS PART OF A REVIEW PROCEDURE FOR PUBLICATION, IT SHOULD NOT REPLACE THE EXPERTISE OF AN EXPERIENCED CRYSTALLOGRAPHIC REFEREE.

No syntax errors found.   CIF dictionary   Interpreting this report

Datablock: ws09a1_gaussian

```
Bond precision:  C-C = 0.0044 A          Wavelength=1.54184

Cell:           a=8.37264(5)    b=10.80933(9)      c=9.22608(7)
                alpha=90        beta=98.2372(6)    gamma=90
Temperature:    100 K

                Calculated                Reported
Volume          826.370(11)               826.371(10)
Space group     P 21                      P 1 21 1
Hall group      P 2yb                     P 2yb
Moiety formula  C19 H16 I N O             C19 H16 I N O
Sum formula     C19 H16 I N O             C19 H16 I N O
Mr              401.23                    401.23
Dx,g cm-3       1.612                     1.612
Z               2                         2
Mu (mm-1)       15.222                    15.222
F000            396.0                     396.0
F000'           396.41
h,k,lmax        10,13,11                  10,13,11
Nref            3380[ 1784]               3356
Tmin,Tmax       0.127,0.107               0.117,0.316
Tmin'           0.017

Correction method= # Reported T Limits: Tmin=0.117 Tmax=0.316
AbsCorr = GAUSSIAN

Data completeness= 1.88/0.99      Theta(max)= 74.415

R(reflections)= 0.0145( 3352)     wR2(reflections)= 0.0384( 3356)

S = 1.090                  Npar= 200
```

The following ALERTS were generated. Each ALERT has the format
test-name_ALERT_alert-type_alert-level.
Click on the hyperlinks for more details of the test.

Alert level G

```
PLAT142_ALERT_4_G s.u. on b - Axis Small or Missing .............    0.00009 Ang.
PLAT143_ALERT_4_G s.u. on c - Axis Small or Missing .............    0.00007 Ang.
PLAT431_ALERT_2_G Short Inter HL..A  Contact I1     ..  O1      ..      2.90 Ang.
PLAT791_ALERT_4_G The Model has Chirality at C2      (Chiral SPGR)         R Verify
PLAT913_ALERT_3_G Missing # of Very Strong Reflections in FCF ....         1 Note
PLAT961_ALERT_5_G Dataset Contains no Negative Intensities .......     Please Check
PLAT978_ALERT_2_G Number C-C Bonds with Positive Residual Density.        12 Note
```

---

0 **ALERT level A** = Most likely a serious problem - resolve or explain
0 **ALERT level B** = A potentially serious problem, consider carefully
0 **ALERT level C** = Check. Ensure it is not caused by an omission or oversight
7 **ALERT level G** = General information/check it is not something unexpected

0 ALERT type 1 CIF construction/syntax error, inconsistent or missing data
2 ALERT type 2 Indicator that the structure model may be wrong or deficient
1 ALERT type 3 Indicator that the structure quality may be low
3 ALERT type 4 Improvement, methodology, query or suggestion
1 ALERT type 5 Informative message, check

It is advisable to attempt to resolve as many as possible of the alerts in all categories. Often the minor alerts point to easily fixed oversights, errors and omissions in your CIF or refinement strategy, so attention to these fine details can be worthwhile. In order to resolve some of the more serious problems it may be necessary to carry out additional measurements or structure refinements. However, the purpose of your study may justify the reported deviations and the more serious of these should normally be commented upon in the discussion or experimental section of a paper or in the "special_details" fields of the CIF. checkCIF was carefully designed to identify outliers and unusual parameters, but every test has its limitations and alerts that are not important in a particular case may appear. Conversely, the absence of alerts does not guarantee there are no aspects of the results needing attention. It is up to the individual to critically assess their own results and, if necessary, seek expert advice.

Publication of your CIF in IUCr journals

A basic structural check has been run on your CIF. These basic checks will be run on all CIFs submitted for publication in IUCr journals (*Acta Crystallographica*, *Journal of Applied Crystallography*, *Journal of Synchrotron Radiation*); however, if you intend to submit to *Acta Crystallographica Section C* or *E* or *IUCrData*, you should make sure that full publication checks are run on the final version of your CIF prior to submission.

Publication of your CIF in other journals

Please refer to the *Notes for Authors* of the relevant journal for any special instructions relating to CIF submission.

PLATON version of 24/11/2016; check.def file version of 23/11/2016

Datablock ws09a1_gaussian - ellipsoid plot

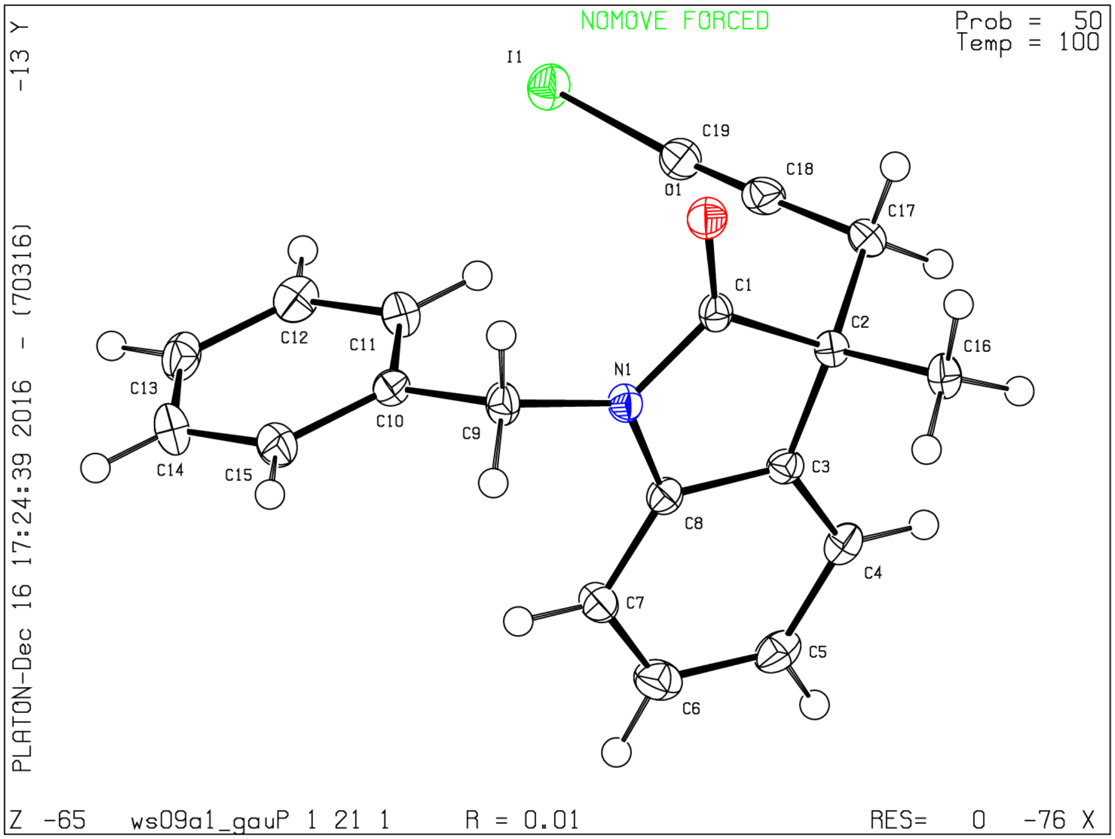

Supplement: Supplementary file 5 — Supplementary Information [file 41598_2019_50940_MOESM5_ESM.pdf]
